# Magnetic Resonance Spectroscopic Imaging and Volumetric Measurements of the Brain in Patients with Postcancer Fatigue: A Randomized Controlled Trial

**DOI:** 10.1371/journal.pone.0074638

**Published:** 2013-09-11

**Authors:** Hetty Prinsen, Arend Heerschap, Gijs Bleijenberg, Machiel J. Zwarts, Jan Willem H. Leer, Jack J. van Asten, Marinette van der Graaf, Mark Rijpkema, Hanneke W. M. van Laarhoven

**Affiliations:** 1 Department of Medical Oncology, Radboud University Nijmegen Medical Centre, Nijmegen, Gelderland, The Netherlands; 2 Department of Radiology, Radboud University Nijmegen Medical Centre, Nijmegen, Gelderland, The Netherlands; 3 Expert Centre for Chronic Fatigue, Radboud University Nijmegen Medical Centre, Nijmegen, Gelderland, Noord-Brabant, The Netherlands; 4 Epilepsy Centre Kempenhaeghe, Heeze, The Netherlands; 5 Department of Radiation Oncology, Radboud University Nijmegen Medical Centre, Nijmegen, Gelderland, The Netherlands; 6 Department of Pediatrics, Radboud University Nijmegen Medical Centre, Nijmegen, Gelderland, The Netherlands; 7 Nuclear Medicine, Radboud University Nijmegen Medical Centre, Nijmegen, Gelderland, The Netherlands; 8 Department of Medical Oncology, Academic Medical Center, University of Amsterdam, Amsterdam, Noord-Holland, The Netherlands; Northwestern University Feinberg School of Medicine, United States of America

## Abstract

**Background:**

Postcancer fatigue is a frequently occurring problem, impairing quality of life. Until now, little is known about (neuro) physiological factors determining postcancer fatigue. For non-cancer patients with chronic fatigue syndrome, certain characteristics of brain morphology and metabolism have been identified in previous studies. We investigated whether these volumetric and metabolic traits are a reflection of fatigue in general and thus also of importance for postcancer fatigue.

**Methods:**

Fatigued patients were randomly assigned to either the intervention condition (cognitive behavior therapy) or the waiting list condition. Twenty-five patients in the intervention condition and fourteen patients in the waiting list condition were assessed twice, at baseline and six months later. Baseline measurements of 20 fatigued patients were compared with 20 matched non-fatigued controls. All participants had completed treatment of a malignant, solid tumor minimal one year earlier. Global brain volumes, subcortical brain volumes, metabolite tissue concentrations, and metabolite ratios were primary outcome measures.

**Results:**

Volumetric and metabolic parameters were not significantly different between fatigued and non-fatigued patients. Change scores of volumetric and metabolic parameters from baseline to follow-up were not significantly different between patients in the therapy and the waiting list group. Patients in the therapy group reported a significant larger decrease in fatigue scores than patients in the waiting list group.

**Conclusions:**

No relation was found between postcancer fatigue and the studied volumetric and metabolic markers. This may suggest that, although postcancer fatigue and chronic fatigue syndrome show strong resemblances as a clinical syndrome, the underlying physiology is different.

**Trial Registration:**

ClinicalTrials.gov NCT01096641

## Introduction

One of the well-known problems of patients undergoing cancer treatment is fatigue [1,2]. According to longitudinal studies, about 20 to 40% of the cancer survivors suffer from persistent fatigue, sometimes even years after successful completion of cancer treatment [3–7]. Postcancer fatigue is a severe and invalidating problem, impairing quality of life [8,9]. Cognitive behavior therapy (CBT) addresses the perpetuating factors of postcancer fatigue and has a clinically relevant effect on reducing fatigue in severely fatigued cancer survivors [10]. However, until now, little is known about (neuro) physiological factors determining postcancer fatigue.

For non-cancer patients with chronic fatigue syndrome (CFS), certain characteristics of brain morphology and metabolism have been identified in previous studies. Using magnetic resonance imaging (MRI) and voxel based morphometry, in a study of 16 CFS patients and 49 healthy controls and in another study of 28 CFS patients and 28 healthy controls, significantly reduced gray matter volumes were observed in CFS patients compared to controls [11,12]. Interestingly, it has been shown that CBT led to a significant increase in gray matter volume in CFS patients [13]. These findings indicate that the cerebral atrophy associated with CFS can be partially reversed after effective CBT [13].

In addition, altered levels of specific metabolites have been reported in the brains of CFS patients, measured with magnetic resonance spectroscopy (MRS). In a study of 7 CFS patients and 10 healthy controls, a significantly reduced level of N-acetylaspartate (NAA) was observed in the hippocampus of CFS patients compared to controls [14]. This result has been attributed to a reduction in neuronal and/or glial cell density or metabolism, as NAA is a marker of neuronal and axonal integrity [15]. The hippocampus plays a pivotal role both in working memory and in long-term memory storage and retrieval, which could be related to the reduced ability of CFS patients to perform memory tasks [16].

In another MRS study of 8 CFS patients and 8 healthy controls, the mean ratio of choline (Cho) to creatine (Cr) in the occipital cortex was demonstrated to be significantly higher in CFS patients compared to controls [17]. As creatine tends to be relatively stable, this result suggests that choline is increased in the occipital cortex of CFS patients. Increased choline levels are associated with abnormal cell membrane metabolism [15]. It has been hypothesized that brain metabolites in the frontal and occipital cortex are altered in CFS patients, because simple reaction times are longer in CFS patients than in controls [18], and simple reaction times might reflect the functioning of both the frontal and occipital lobes [19].

It may be hypothesized that these metabolic and volumetric traits found in CFS patients are a reflection of fatigue in general and thus may also be of importance for patients suffering from postcancer fatigue. In this study we extended the survey of metabolites in the hippocampus with the gliosis-associated marker myoinositol (mI) and the excitatory neurotransmitter glutamate (Glu) and the metabolite ratios mI: NAA and Glu: mI [20,21]. Next to global volumes of gray and white matter, as has been studied in CFS patients, we examined subcortical brain volumes.

The aims of this study were A) to examine if volumetric and metabolic parameters are different between severely fatigued and non-fatigued cancer survivors and B) to examine the effect of CBT on these volumetric and metabolic markers in severely fatigued cancer survivors.

## Materials and Methods

The protocol for this trial and supporting CONSORT checklist are available as supporting information; see Checklist S1 and Protocol S1.

### Trial registration

The study is registered at ClinicalTrials.gov (NCT01096641).

### Participants

**Figure 1:**
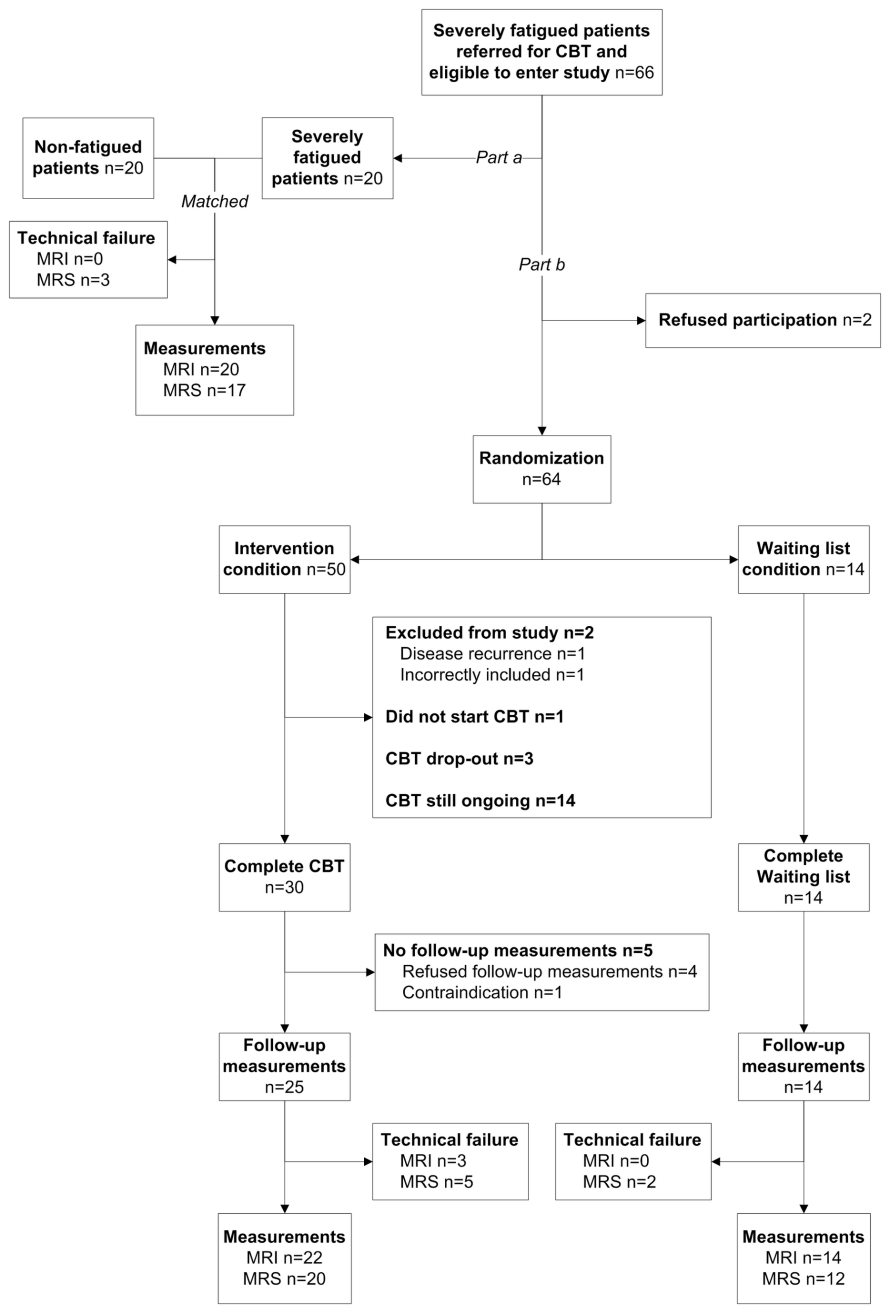
Flow chart of the study.

The local ethics committee of the Radboud University Nijmegen Medical Centre (RUNMC, Nijmegen, the Netherlands) approved the study and all participants provided written informed consent. In part A of the study, severely fatigued and non-fatigued cancer survivors were compared (Figure 1). In part B of the study, severely fatigued cancer survivors were randomly assigned to either the intervention condition or the waiting list condition (Figure 1). Fatigue severity was measured by the fatigue severity subscale of the Checklist Individual Strength (CIS-fatigue) [22,23]. Severe fatigue was defined by a cut-off score of ≥35points [7,10,24,25]. The CIS-fatigue has been used in previous research investigating fatigue in cancer patients and was shown to be sensitive to detect changes.

All participants had completed curative treatment of a malignant, solid tumor or of a (non-) Hodgkin’s lymphoma a minimum of 1 year earlier, and had no evidence of disease recurrence at the time of the study. The minimum age of disease onset was 18 years, patients were no older than 65 years of age when entering the study. Patients had no current psychological or psychiatric treatment and used no anti-depressive drugs, anti-epileptic drugs, or benzodiazepines when participating in the study. Patients had no brain tumor in the past and had no physical comorbidity (e.g. anemia, poor kidney function, etc.) that could explain fatigue.

For the evaluation of severely fatigued cancer survivors, referred for CBT to the Expert Centre for Chronic Fatigue of the RUNMC, versus non-fatigued cancer survivors (part A of the study), patients were included from March 2009 onwards. Enrollment of severely fatigued cancer survivors for the parallel-group randomized controlled trial (part B of the study) continued in March 2010 until April 2012. Sixty-four fatigued patients consented and were randomly assigned at a ratio of 3:1 to either the intervention condition (n=50) or the waiting list condition (n=14). Random assignment was done by means of a sequence of labeled cards contained in sealed, numbered envelopes prepared by a statistical adviser. The envelopes were opened by the psychologists in the presence of the patient. Patients randomized to the intervention group were immediately treated with CBT for postcancer fatigue, as described previously [10], whereas patients randomized to the control group waited six months for CBT and received CBT outside the study. Both the intervention and the waiting list group were assessed twice, at baseline and at six months follow-up, at the RUNMC. Data are presented of patients who completed both the baseline and follow-up measurements.

Baseline measurements of 20 of the 64 randomized severely fatigued cancer survivors were compared with 20 age- and sex-matched non-fatigued patients, recruited from the outpatient clinics of Medical Oncology and Radiation Oncology of the RUNMC (part A of the study). Non-fatigued patients were assessed only once, at the RUNMC.

### Measurements

MRI and MRS measurements were performed on a 3 Tesla MR system (Tim TRIÓ, Siemens, Erlangen, Germany) using the standard circularly polarized birdcage head coil. Fatigue scores, global brain volumes, subcortical brain volumes, metabolite tissue concentrations, and metabolite ratios were the primary outcome measures.

#### Volumetric measurements

High-resolution 3D T1-weighted anatomical images of the whole brain (voxel size 1x1x1mm^3^) were acquired using a magnetization prepared rapid acquisition gradient echo sequence (TR=2300 ms, TE=3.16 ms). Raw MRI data in DICOM format were converted to NIFTI format using the conversion as implemented in the SPM5 package (http://www.fil.ion.ucl.ac.uk/spm/software/spm5/).

Normalizing, bias-correcting, and segmentation of gray matter (GM) and white matter (WM) was performed using the voxel-based morphometry (VBM) toolbox (VBM5.1 Toolbox version 1.19) in SPM5 using priors (default settings). This method uses an optimized VBM protocol [26,27] as well as a model based on Hidden Markov Random Fields (HMRF) developed to increase signal-to-noise ratio [28]. Total GM volume (GMV) and WM volume (WMV) were calculated by adding the resulting tissue probabilities. Total brain volume (TBV) was defined as the sum of WMV and GMV.

Automatic segmentation of subcortical brain structures was performed using the FIRST module (version 1.1) of FSL (version 4.1.4) (http://www.fmrib.ox.ac.uk/fsl) [29,30]. Volumetry was applied to segment brainstem (defined as medulla, pons, and midbrain, bordering the ventral diencephalon, the fourth ventricle, and the cerebellum) and bilateral accumbens, amygdala, caudate nucleus, hippocampus, globus pallidus, putamen, and thalamus. Volumes of bilateral structures were added-up. Subcortical brain volumes were expressed as a percentage of TBV.

#### ^1^H Magnetic Resonance Spectroscopic Imaging

The T1-weighted images were used to position one 2D MR spectroscopic image (MRSI) slice in the plane perpendicular to the longitudinal axis of the hippocampus and a second 2D MRSI slice in the plane including the occipital cortex. Volume selection was done by using the semi-LASER pulse sequence [31,32] with 12x16 phase encoding steps for MRSI in the hippocampus and 16x20 phase encoding steps for MRSI in the occipital cortex (nominal voxel size 10.0x10.0x10.0 mm, TR=1500 ms, TE=30 ms for the hippocampus, TE=136 ms for the occipital cortex, 6 acquisition-weighted averages). MRSI data of the hippocampus were acquired with and without water signal suppression for referencing purposes [32]. Metabolite tissue concentrations were obtained from two voxels selected in the hippocampus (one voxel in the right and one voxel in the left hippocampus) and from two voxels selected in the occipital cortex adjacent to each other, close to the parieto-occipital sulcus. LCModel was used to obtain absolute tissue concentrations (water referenced, corrected for T1 and T2 relaxation, but not for cerebrospinal fluid contribution) of NAA, mI, and Glu, and the concentration ratios of mI: NAA, and Glu: mI in the hippocampus. Signal ratios of Cho: Cr in the occipital cortex were also obtained using LCModel. Per subject, the metabolite tissue concentrations, metabolite concentration ratios, and metabolite signal ratios were calculated for the two voxels per location and subsequently averaged.

### Statistics

Statistical analyses were performed using PASW for Windows®, version 18.0.2 (Armonk, New York, USA). Results are presented as absolute numbers, as mean ± standard deviation (SD), as frequencies with percentages of total, or as change scores (the percentage change from baseline to follow-up). Normality of the data was tested using a Shapiro-Wilk test. Chi square tests, independent samples *t* tests, and Mann–Whitney U tests were performed to compare differences in baseline characteristics between fatigued and non-fatigued patients and between the therapy and the waiting list condition (Table 1). To compare differences in MR results between fatigued and sex- and age-matched non-fatigued cancer survivors, independent samples *t* tests were performed (Table 2). Given the differences in brain size and morphology between men and women [33] and the age-related changes in GM [11,27], the normally distributed data of Table 3 are corrected for sex and age by analyses of covariance. Differences in the non-normally distributed parameters between the therapy and the waiting list condition were tested by Mann–Whitney U tests (Table 3). To evaluate the uncontrolled within group effects of CGT and WL from baseline to follow-up, paired *t* tests were performed for the normally distributed parameters and the Wilcoxon matched-pairs tests for the non-normally distributed parameters (Table 4). Differences were considered statistically significant at *p*<0.05. The power calculation is described in the study protocol as published previously [34]. As a result of financial and logistic reasons, it was not possible to perform the follow-up measurements of the patients still undergoing CBT.

**Table 1:** Baseline characteristics of fatigued and non-fatigued patients (part A of the study) and of fatigued patients in the therapy and waiting list condition (part B of the study).

	**Fatigued (n=20**)	**Non-fatigued (n=20**)	***p* value**	**CBT (*n*=25**)	**WL (*n*=14**)	***p* value**
**Male / Female (n**)	10/10	10/10	1.000	14 / 11	5 / 9	0.224
**Age (years**)	47.9 ± 10.1	48.9 ± 9.7	0.751	48.8 ± 9.4	50.6 ± 10.9	0.583
**Time since cancer treatment (months**)	64.4 ± 69.7	60.1 ± 44.0	0.580	55.0 ± 61.7	44.4 ± 36.4	0.372
**Cancer diagnosis (n**)						
Breast cancer	6 (30)	9 (45)		8 (32)	6 (43)	
Head and neck cancer	3 (15)	2 (10)		6 (24)	2 (14)	
Testicular cancer	2 (10)	3 (15)		3 (12)	0	
(Non)Hodgkin	2 (10)	2 (10)		2 (8)	3 (21)	
Prostate cancer	2 (10)	0		2 (8)	1 (7)	
Thyroid cancer	2 (10)	0		2 (8)	0	
Other solid cancers	3 (15)	4 (20)		2 (8)	2 (14)	
**Cancer treatment (n**)						
Surgery only	2 (10)	1 (5)		3 (12)	1 (7)	
Surgery and CT	5 (25)	4 (20)		6 (24)	1 (7)	
Surgery and RT	2 (10)	2 (10)		2 (8)	2 (14)	
Surgery and RI	1 (5)	0		2 (8)	0	
Surgery and IT	0	1 (5)		0	1 (7)	
Surgery, RT and CT	4 (20)	3 (15)		4 (16)	1 (7)	
Surgery, RT, and HT	1 (5)	0		1 (4)	0	
Surgery, RT, and RI	1 (5)	0		1 (4)	0	
Surgery, CT, and HT	0	2 (10)		2 (8)	2 (14)	
Surgery, RT, CT, and HT	2 (10)	4 (20)		2 (8)	3 (21)	
CT only	1 (5)	1 (5)		0	1 (7)	
CT and RT	1 (5)	1 (5)		1 (4)	1 (7)	
RT only	0	1 (5)		0	1 (7)	

Data are presented as absolute numbers, as mean ± standard deviation, or as frequencies with percentages in brackets. Independent samples *t* tests (age), chi square tests (sex), and Mann Whitney-U tests (time since cancer treatment) were performed. Abbreviations: CBT: cognitive behavior therapy; CT: chemotherapy; HT: hormonal therapy; IT: immunotherapy; RI: radioactive iodine; RT, radiotherapy; WL: waiting list.

**Table 2:** MR results of fatigued and non-fatigued patients.

	**Fatigued (n=20**)	**Non-fatigued (n=20**)	***p* value**
**Global brain volumes**	**(n=20**)	**(n=20**)	
Gray matter volume (ml)	689.1 ± 76.8	661.6 ±60.4	0.449
White matter volume (ml)	530.8 ± 94.3	501.3 ± 62.4	0.703
Total brain volume (ml)	1219.9 ±153.3	1162.9 ±111.7	0.703
**Subcortical brain volumes**	**(n=20**)	**(n=20**)	
Accumbens (% of total brain volume)	0.090 ± 0.013	0.097 ± 0.018	0.150
Amygdala (% of total brain volume)	0.280 ± 0.043	0.299 ± 0.040	0.155
Caudate nucleus (% of total brain volume)	0.606 ± 0.054	0.596 ± 0.053	0.578
Hippocampus (% of total brain volume)	0.647 ± 0.063	0.679 ± 0.065	0.113
Globus pallidus (% of total brain volume)	0.321 ± 0.024	0.319 ± 0.031	0.783
Putamen (% of total brain volume)	0.873 ± 0.062	0.891 ± 0.065	0.360
Thalamus (% of total brain volume)	1.318 ± 0.077	1.357 ± 0.073	0.108
Brainstem (% of total brain volume)	1.911 ± 0.130	1.957 ± 0.139	0.291
**Metabolite signal ratios occipital cortex**	**(n=17**)	**(n=17**)	
Choline:Creatine	0.34 ± 0.07	0.37 ± 0.06	0.245
**Metabolite tissue levels and concentration ratios hippocampus**	**(n=17**)	(n=17)	
N-acetylaspartate (mmol/l)	8.63 ± 0.72	8.80 ± 0.90	0.539
Myoinositol (mmol/l)	8.25 ± 1.56	7.64 ± 2.21	0.362
Glutamate (mmol/l)	7.31 ± 0.97	6.69 ± 1.32	0.128
Myoinositol:N-acetylaspartate	0.97 ± 0.23	0.87 ± 0.26	0.275
Glutamate:Myoinositol	0.92 ± 0.23	0.95 ± 0.32	0.742

Data are presented as mean ± standard deviation. Independent samples *t* tests were performed. Fatigued and non-fatigued patients were matched by sex and age.

**Table 3:** MR results of fatigued patients in the therapy and waiting list condition, presented as change scores.

	**CBT (n=25**)	**WL (n=14**)	***p* value**
**Global brain volumes (%**) Gray matter volume	(**n=22**) -0.66 ± 1.39	(**n=14**) -0.41 ± 1.10	0.860
White matter volume	-0.20 ± 0.76	-0.21 ± 0.86	0.713
Total brain volume	-0.47 ± 1.04	-0.32 ± 0.66	0.987
**Subcortical brain volumes (%**)	**(n=22**)	**(n=14**)	
Accumbens	-1.50 ± 8.18	0.23 ± 6.21	0.404
Amygdala	1.60 ± 6.28	2.47 ± 3.35	0.841
Caudate nucleus	0.26 ± 1.78	0.50 ± 2.00	0.688
Hippocampus	-0.25 ± 2.45	-0.22 ± 2.63	0.680
Globus pallidus	0.28 ± 2.71	-0.89 ± 2.23	0.140
Putamen	0.24 ± 1.93	-0.91 ± 2.07	0.114
Thalamus	0.10 ± 1.79	0.79 ± 1.33	0.254
Brainstem	0.37 ± 3.00	-0.97 ± 2.45	0.289
**Metabolite signal ratios occipital cortex (%**)	**(n=20**)	**(n=12**)	
Choline:Creatine	-1.45 ± 40.54	3.60 ± 25.77	0.732
**Metabolite tissue levels and concentration ratios hippocampus (%**)	**(n=20**)	**(n=12**)	
N-acetylaspartate	2.24 ± 10.60	0.32 ± 7.72	0.620
Myoinositol	7.32 ± 30.55	-2.97 ± 23.86	0.305
Glutamate	8.86 ± 28.61	-3.90 ± 21.02	0.233
Myoinositol:N-acetylaspartate	4.89 ± 27.22	-2.71 ± 25.58	0.413
Glutamate:Myoinositol	5.93 ± 32.60	2.02 ± 23.43	0.793

Data are presented as change scores (the percentage change from baseline to follow-up). Analyses of covariance (subcortical brain volumes, metabolite signal ratios occipital cortex, metabolite tissue levels and concentration ratios hippocampus), correcting for age and sex, and Mann Whitney-U tests (global brain volumes) were performed. Abbreviations: CBT: cognitive behavior therapy; WL: waiting list.

**Table 4:** Uncontrolled within group analyses of MR results of fatigued patients in the therapy and waiting list condition from baseline to follow-up.

	**CBT (n=25**)	**CBT (n=25**)		**WL (n=14**)	**WL (n=14**)	
	**Baseline**	**Follow-up**	***p*-value**	**Baseline**	**Follow-up**	***p*-value**
**Global brain volumes** Gray matter volume (ml)	(**n=22**) 686.9 ± 76.2	(**n=22**) 682.3.6 ± 75.7	(**n=22**) 0.074	(**n=14**) 635.5 ± 65.0	(**n=14**) 632.6 ± 61.9	(**n=14**) 0.268
White matter volume (ml)	502.8 ± 85.6	502.0 ± 87.6	0.483	496.4 ± 98.8	495.4 ± 99.2	0.542
Total brain volume (ml)	1189.7 ± 151.9	1184.3 ± 153.8	0.147	1131.9 ± 149.0	1128.0 ± 145.4	0.091
**Subcortical brain volumes**	**(n=22**)	**(n=22**)	**(n=22**)	**(n=14**)	**(n=14**)	**(n=14**)
Accumbens	0.096 ± 0.014	0.095 ± 0.014	0.306	0.096 ± 0.017	0.096 ± 0.017	0.964
Amygdala	0.278 ± 0.028	0.282 ± 0.029	0.275	0.300 ± 0.042	0.307 ± 0.041	0.018
Caudate nucleus	0.620 ± 0.062	0.622 ± 0.065	0.466	0.627 ± 0.052	0.630 ± 0.054	0.366
Hippocampus	0.662 ± 0.053	0.660 ± 0.052	0.581	0.668 ± 0.071	0.667 ± 0.076	0.828
Globus pallidus	0.319 ± 0.023	0.320 ± 0.028	0.536	0.340 ± 0.025	0.337 ± 0.026	0.147
Putamen	0.877 ± 0.062	0.879 ± 0.066	0.546	0.910 ± 0.038	0.902 ± 0.048	0.129
Thalamus	1.328 ± 0.075	1.329 ± 0.074	0.843	1.384 ± 0.095	1.394 ± 0.095	0.049
Brainstem	1.898 ± 0.171	1.921 ± 0.182	0.071	2.033 ± 0.178	2.016 ± 0.134	0.360
**Metabolite signal ratios occipital cortex**	**(n=20**)	**(n=20**)	**(n=20**)	**(n=12**)	**(n=12**)	**(n=12**)
Choline:Creatine	0.37 ± 0.10	0.34 ± 0.09	0.336	0.35 ± 0.10	0.35 ± 0.07	0.860
**Metabolite tissue levels and concentration ratios hippocampus**	**(n=20**)	**(n=20**)	**(n=20**)	**(n=12**)	**(n=12**)	**(n=12**)
N-acetylaspartate	8.80 ± 0.93	8.95 ± 0.91	0.476	8.92 ± 0.63	8.92 ± 0.65	0.981
Myoinositol	7.93 ± 1.74	8.22 ± 1.74	0.537	8.35 ± 1.32	7.96 ± 1.76	0.498
Glutamate	7.08 ± 1.24	7.44 ± 1.11	0.360	7.43 ± 0.73	7.05 ± 1.28	0.422
Myoinositol:N-acetylaspartate	0.92 ± 0.25	0.92 ± 0.20	0.893	0.94 ± 0.14	0.89 ± 0.19	0.503
Glutamate:Myoinositol	0.93 ± 0.24	0.95 ± 0.28	0.733	0.91 ± 0.16	0.92 ± 0.22	0.895

Data are presented as mean ± standard deviation. *P*-values of uncontrolled within group analyses from baseline to follow-up are presented for the therapy and waiting list condition. Paired *t* tests (subcortical brain volumes, metabolite signal ratios occipital cortex, metabolite tissue levels and concentration ratios hippocampus) and Wilcoxon matched-pairs tests (global brain volumes) were performed. Abbreviations: CBT: cognitive behavior therapy; WL: waiting list.

## Results

### Patient inclusion

The flow chart of the study is presented in Figure 1. Both severely fatigued and non-fatigued cancer survivors were enrolled in this study between March 2009 and April 2012 and the last follow-up measurements were performed in September 2012. Of the 66 severely fatigued patients who were referred for CBT to the Expert Centre for Chronic Fatigue and who met the criteria of the study, baseline data of 20 patients were compared with data of 20 age- and sex-matched non-fatigued patients (part A of the study). Two severely fatigued patients refused CBT and, therefore, did not participate in the randomized controlled trial (part B of the study). Of the 64 patients who were randomized, 50 patients were allocated to the intervention condition and 14 patients were allocated to the waiting list condition. After randomization, one patient had disease recurrence, one patient was incorrectly included according to the inclusion criteria, one patient did not want to start CBT, and three patients dropped out during CBT. As a result of financial and logistic reasons, it was not possible to perform the follow-up measurements of the 14 patients still undergoing CBT. The patients still undergoing CBT were not included in the analyses.

Twenty-five patients completed both the baseline and follow-up measurements in the intervention condition and 14 patients in the waiting list condition. Due to technical failure, 3 MRI and 5 MRS measurements in the intervention condition could not be analyzed and 2 MRS measurements in the waiting list condition failed.

### Study population

Part A) Baseline characteristics of the 20 fatigued and the 20 sex- and age-matched non-fatigued cancer survivors are presented in Table 1. Time since cancer treatment did not significantly differ between the patients suffering from fatigue and the non-fatigued group. Breast cancer was the most common cancer type. Ninety percent and 85% of the fatigued and non-fatigued participants, respectively, underwent surgery.

Part B) Baseline characteristics of the 25 fatigued patients in the therapy condition and the 14 patients in the waiting list condition are presented in Table 1. Sex, age, time since cancer treatment, and fatigue severity were similar in the intervention and the waiting list group. Breast cancer was the most common cancer type. Ninety-six percent and 79% of the patients in the therapy and the waiting list condition, respectively, underwent surgery.

At baseline, no significant differences were present in age, sex, time since cancer treatment, and fatigue severity between the 25 patients in the intervention condition, who completed both the baseline and follow-up measurements, and the 25 patients in the intervention condition, for whom baseline measurements were available only (data not shown).

### Part A. Fatigued versus non-fatigued patients

**Figure 2:**
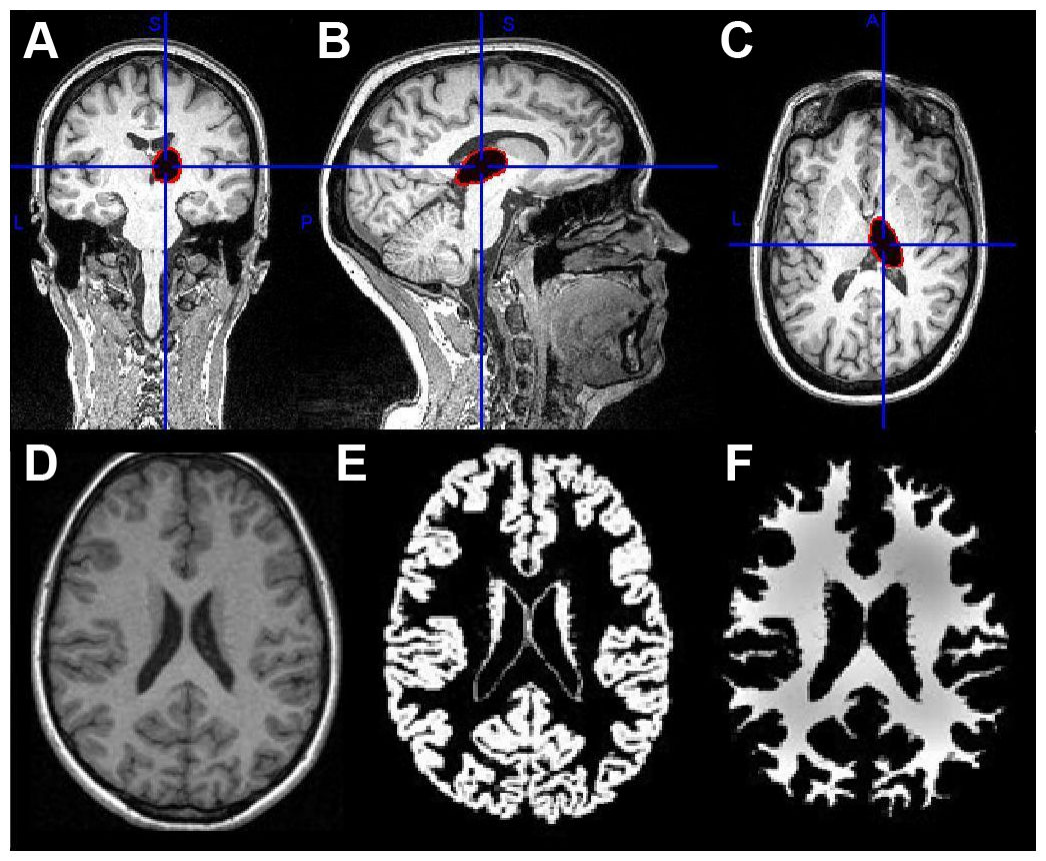
Examples of subcortical and global brain segmentation. An example of subcortical brain segmentation of the thalamus in red in coronal (A), sagittal (B), and transversal plane (C), and an example of voxel-based segmentation of an anatomical image (D) in a gray matter image (E) and a white matter image (F).

**Figure 3:**
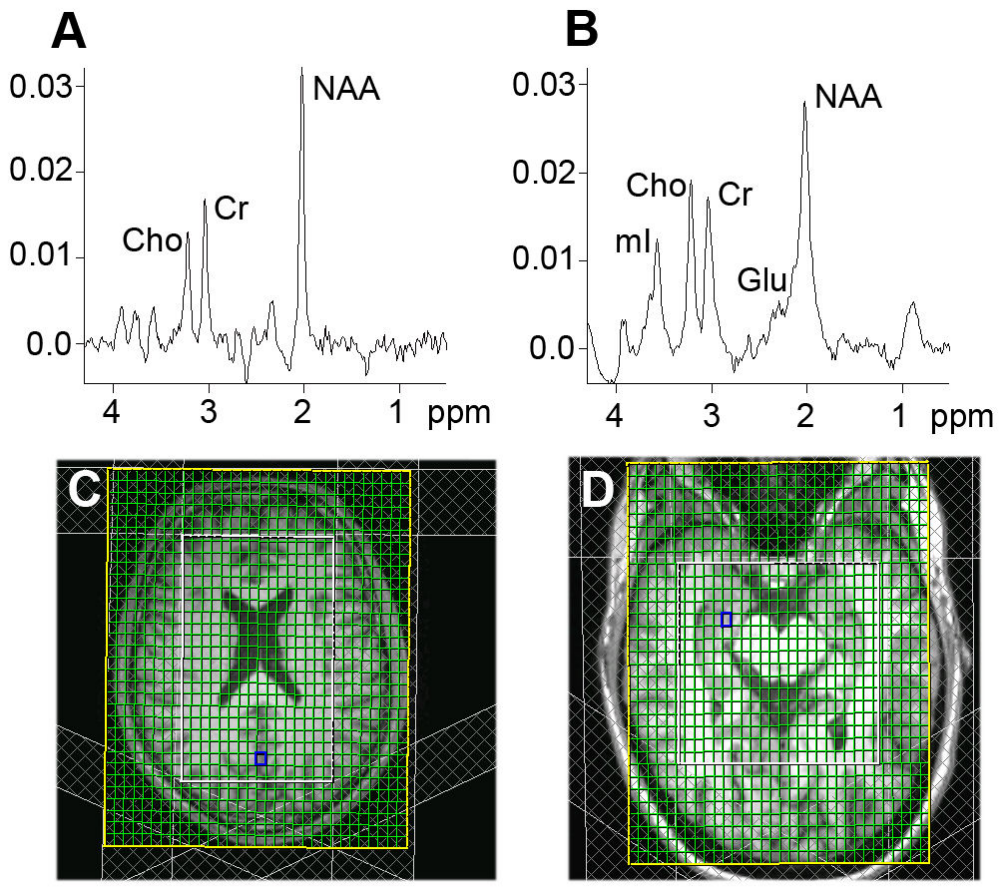
Examples of spectra, background images, and ^1^H Magnetic Resonance Spectroscopic Imaging grid. An example of a Hanning filtered spectrum in the occipital cortex (A) and in the hippocampus (B) and the accompanying background image plus ^1^H Magnetic Resonance Spectroscopic Imaging grid (respectively C and D).

Subcortical brain volumes (accumbens, amygdala, caudate nucleus, hippocampus, globus pallidus, putamen, thalamus, and brainstem, Figure 2A–C) and global brain volumes (GMW, WMV, and TBV, Figure 2D–F) were not significantly different between fatigued and non-fatigued patients (Table 2). In the hippocampus, metabolite tissue concentrations (NAA, mI, and Glu) and metabolite concentration ratios (mI: NAA and Glu: mI) did not significantly differ between fatigued and non-fatigued patients (Table 2 and Figure 3). Finally, metabolite signal ratios in the occipital cortex (Cho: Cr) were not significantly different between fatigued and non-fatigued patients (Table 2 and Figure 3).

### Part B. Cognitive behavior therapy versus waiting list

After 6 months follow-up, patients who underwent CBT, with a mean of 11.8±5.1 individual sessions, reported a significantly larger change in fatigue scores than patients who waited 6 months for CBT (*p*<0.001, respectively -45.5±22.7% and -16.4±25.0%).

Change scores (percentage change from baseline to follow-up) of global brain volumes (GMW, WMV, and TBV) and subcortical brain volumes (accumbens, amygdala, caudate nucleus, hippocampus, globus pallidus, putamen, thalamus, and brainstem) were not significantly different between patients in the therapy group and patients in the waiting list group (Table 3). In the hippocampus, metabolite tissue concentrations (NAA, mI, and Glu) and metabolite concentration ratios (mI: NAA and Glu: mI) were not significantly different between patients in the therapy group and patients in the waiting list group (Table 3). Also, metabolite signal ratios in the occipital cortex (Cho: Cr) did not significantly differ between patients in the therapy and patients in the waiting list group (Table 3).

Uncontrolled within group analyses showed no significant effect of CBT from baseline to follow-up on global brain volumes, subcortical brain volumes, metabolite tissue concentrations, metabolite concentration ratios, and metabolite signal ratios (Table 4). The WL condition showed also no effect from baseline to follow-up on these volumetric and metabolic parameters, except for a significant increase in the volume of the amygdala and the thalamus (Table 4).

## Discussion

To the best of our knowledge, this is the first study to report on volumetric and metabolic parameters in the brain of severely fatigued and non-fatigued disease-free cancer survivors. In contrast to findings in CFS, no volumetric and metabolic differences between fatigued and non-fatigued cancer survivors were observed. In addition, this is the first randomized controlled trial examining the effect of CBT on volumetric and metabolic markers in severely fatigued disease-free cancer survivors. CBT for fatigue in cancer survivors is an effective therapy, as has been shown before [10]. In our study CBT also resulted in a significantly larger decrease in fatigue severity compared to a period of waiting for therapy. However, we did not observe significant effects of CBT on volumetric or metabolic markers in the brain.

In the study of de Lange et al. [13], gray matter volume was significantly smaller in CFS patients (669.4 ± 14.4 ml) compared to healthy controls (708.2 ± 12.0 ml) and CFS patients showed a significant increase in gray matter volume from pre-CBT to post-CBT (674.1 ± 15.1 ml). Although not significantly different between fatigued and non-fatigued cancer survivors, the gray matter volumes were comparable to the CFS study. In fatigued patients, gray matter volume did not significantly change from pre- to post-CBT or from pre- to post-waiting list. In both studies, anatomical images were acquired using a magnetization prepared rapid acquisition gradient echo sequence and images were analyzed using VBM. The duration of fatigue complaints was comparable in the CFS study (CFS duration in years in CFS patients 5.8 ± 0.79) and the present study (time since cancer treatment in years in fatigued cancer survivors 5.4 ± 5.8). The CFS study was performed at 1.5 Tesla, whereas the present study was performed on a 3.0 Tesla scanner with a better signal-to-noise ratio. Although we did not find significant differences in global brain volumes in the present study, the VBM method is able to demonstrate significant differences between groups, as has been shown before [11–13].

We extended the survey of global brain volumes and examined subcortical brain volumes, but found no associations with postcancer fatigue. This analysis method has shown the potential to detect small differences in subcortical brain volumes, however, in larger groups of subjects [29,35].

The absolute and relative tissue levels of the metabolites determined in this study for non-fatigued patients are comparable with values commonly found for the hippocampus and occipital cortex in normal persons [32,36–38]. However, the decreased levels of NAA in the hippocampus, as found in CFS patients [14], were not observed in our patients with postcancer fatigue. This CFS study concerned far less patients than in our study. It was performed on a 1.5 Tesla MR system and the spectra were acquired using a STEAM sequence, whereas the present study was performed on a 3.0 Tesla MR system and used a semi-LASER sequence, which results in a much better signal-to-noise ratio. Moreover, the T2 relaxation times of the individual metabolites measured at three different echo times in the CFS study are susceptible to small noise contributions, a rather high absolute NAA concentration was reported for healthy controls, and the levels of all main metabolites seemed to decrease for CFS patients compared to healthy controls.

We also could not observe a difference in the Cho: Cr ratio for postcancer fatigues patients as seen in the occipital cortex in another study of CFS patients [17]. This CSF study also concerned a much smaller patient group and was performed at 1.5 Tesla, but further described settings were comparable to the current investigation. An increased ratio of Cho: Cr was also observed in the basal ganglia of another small group of CFS patients [39].

Furthermore, no significant differences could be found between fatigued and non-fatigued cancer survivors for any other investigated metabolite level, such as the Glu tissue concentrations in the hippocampus.

Altogether, the results of our study could not confirm the hypothesis that metabolic and volumetric traits observed in CFS patients are a reflection of fatigue in general and are, therefore, also of importance for patients suffering from postcancer fatigue. This suggests either that, although postcancer fatigue and CFS show strong resemblances as a clinical syndrome, the underlying physiology is different, or that technical differences between the present study in postcancer fatigue and the previous studies in CFS patients explain the differences in study outcomes between both fatigue syndromes. The more likely reason for the observed differences between postcancer fatigue and CFS in metabolite concentrations and ratios may lie in the technical arena, in particular because of the much smaller group sizes and more unfavorable measurement conditions (e.g. lower magnetic fields) in the CFS studies. A direct comparison of the data of the present postcancer fatigue study with the data acquired in a CFS study with the same technical parameters and the same sample size is necessary to address this discrepancy.

Postcancer fatigue appears to be unrelated to abnormalities in brain structure or brain metabolite concentrations, but our results do not imply that alterations in the pathophysiology of the brain can be excluded as underlying mechanism of postcancer fatigue. In particular, alterations in the dynamics of brain physiology, which are not reflected in the static levels of the brain metabolites or in the brain volumes as investigated in this study may be involved. To address if these aspects of brain physiology contribute to the experience of fatigue, it is needed to perform more functional studies such as, for example, resting state functional MRI [40].

### Limitations of the study

We could have investigated more areas in the brain for metabolic differences and the number of patients might still have been relatively small, although the group size was much larger than in comparable studies. Also, the power of the study was sufficiently high to demonstrate a significantly larger decrease in CIS-fatigue score in patients in the therapy group compared to patients in the waiting list group.

In conclusion, no relation was observed between postcancer fatigue and a set of volumetric and metabolic markers in the brain in the present study. Therefore, based on the VBM method to calculate global brain volumes, the FIRST module of FSL to calculate subcortical brain volumes, and ^1^H MRSI to calculate metabolite concentrations and ratios in the hippocampus and the occipital cortex, postcancer fatigue does not appear to be associated with abnormalities in brain structures or brain metabolism. Additional investigations, including functional brain studies, may be needed to identify neurophysiological factors which may explain postcancer fatigue.

## Supporting Information

Checklist S1 **CONSORT Checklist.**
(DOC)

Protocol S1 **Trial Protocol.**
(DOC)
