# Supplementary material for: Magnetic Resonance Spectroscopic Imaging and Volumetric Measurements of the Brain in Patients with Postcancer Fatigue: A Randomized Controlled Trial
Source: PLoS One. 2013 Sep 11;8(9):e74638. doi: 10.1371/journal.pone.0074638 (PMC3770709; doi:10.1371/journal.pone.0074638)
Supplement: Protocol S1 — Trial Protocol. (DOC) [file pone.0074638.s002.doc]

**Physiological and neurophysiological determinants of postcancer fatigue: design of a randomized controlled trial**

Hetty Prinsen^1^, Gijs Bleijenberg^2^, Machiel J. Zwarts^3^, Maria T.E. Hopman^4^, Arend Heerschap^5^, and Hanneke W.M. van Laarhoven^1,6^

^1^Department of Medical Oncology, Radboud University Nijmegen Medical Centre, Nijmegen, the Netherlands, ^2^Expert Centre for Chronic Fatigue, Radboud University Nijmegen Medical Centre, Nijmegen, the Netherlands, ^3^Department of Clinical Neurophysiology, Radboud University Nijmegen Medical Centre, Nijmegen, the Netherlands, ^4^Department of Physiology, Radboud University Nijmegen Medical Centre, Nijmegen, the Netherlands, ^5^Department of Radiology, Radboud University Nijmegen Medical Centre, Nijmegen, the Netherlands, ^6^Department of Medical Oncology, Academic Medical Centre, University of Amsterdam, Amsterdam, the Netherlands.

**Abstract**

**Background:** Postcancer fatigue is a frequently occurring, severe, and invalidating problem, impairing quality of life. Although it is possible to effectively treat postcancer fatigue with cognitive behaviour therapy, the nature of the underlying (neuro)physiology of postcancer fatigue remains unclear. Physiological aspects of fatigue include peripheral fatigue, originating in muscle or the neuromuscular junction; central fatigue, originating in nerves, spinal cord, and brain; and physical deconditioning, resulting from a decreased cardiopulmonary function. Studies on physiological aspects of postcancer fatigue mainly concentrate on deconditioning. Peripheral and central fatigue and brain morphology and function have been studied for patients with fatigue in the context of chronic fatigue syndrome and neuromuscular diseases and show several characteristic differences with healthy controls.

**Methods/Design:** Fifty seven severely fatigued and 21 non-fatigued cancer survivors will be recruited from the Radboud University Nijmegen Medical Centre. Participants should have completed treatment of a malignant, solid tumour minimal one year earlier and should have no evidence of disease recurrence. Severely fatigued patients are randomly assigned to either the intervention condition (cognitive behaviour therapy) or the waiting list condition (start cognitive behaviour therapy after 6 months). All participants are assessed at baseline and the severely fatigued patients also after 6 months follow-up (at the end of cognitive behaviour therapy or waiting list). Primary outcome measures are fatigue severity, central and peripheral fatigue, brain morphology and function, and physical condition and activity.

**Discussion:** This study will be the first randomized controlled trial that characterizes (neuro)physiological factors of fatigue in disease-free cancer survivors and evaluates to which extent these factors can be influenced by cognitive behaviour therapy. The results of this study are not only essential for a theoretical understanding of this invalidating condition, but also for providing an objective biological marker for fatigue that could support the diagnosis and follow-up of treatment.

**Trial registration:** The study is registered at ClinicalTrials.gov (NCT01096641).

**Keywords**

Postcancer fatigue

Cognitive behaviour therapy

Peripheral fatigue

Central fatigue

Brain morphology

Brain metabolism

Physical condition

Physical activity

**Background**

**Postcancer fatigue**

Postcancer fatigue (PCF) is a frequently occurring, severe, and invalidating problem in cancer survivors, impairing quality of life.[1, 2] About 70 to 96 percent of the cancer patients undergoing chemotherapy and/or radiotherapy experience symptoms of fatigue.[3, 4] The prevalence of PCF observed in longitudinal follow-up studies ranged from 19 to 39 percent.[1, 5-8] Previous disease and treatment characteristics are unrelated to PCF.[2, 4, 9-12] However, there is some evidence that patients who are treated with only a surgery are less at risk for PCF[8] and survivors who are treated with more aggressive treatments are more at risk for PCF [1, 13]. Cognitive behaviour therapy (CBT), especially designed for PCF, seemed to be an effective treatment option for PCF.[11, 14] However, although it is now possible to effectively treat PCF, the nature of the underlying physiology of PCF remains unclear.

The term fatigue in the medical literature usually refers to fatigue experienced by the patient, but it can also refer to physiological fatigue. Fatigue experienced by the patient and its psychological aspects can be quantified using questionnaires. Physiological aspects of fatigue include peripheral fatigue, originating in muscle or the neuromuscular junction; central fatigue, originating in nerves, spinal cord, and brain; and physical deconditioning, resulting from a decreased cardiopulmonary function.

**Peripheral and central fatigue**

In physiology, fatigue is usually defined as the loss of voluntary force-producing capacity during exercise,[15] which can have a peripheral and a central origin.[16] During peripheral muscle fatigue, membrane excitability of muscle tissue is influenced due to a decrease in pH, accumulation of lactate, and changes in intra- and extracellular ion concentration.[17] Muscle fibre conduction velocity (MFCV), which can be determined by surface electromyography (sEMG), reflects the peripheral situation.[16] Alternatively, peripheral fatigue can be quantified by measuring the muscular force response to artificial electrical stimulation before and after exercise. Besides peripheral factors, a failure of drive from the central nervous system may also contribute to fatigue[18] This sub-maximal central activation during exercise, or central activation failure (CAF), is named central fatigue[16] and can be measured with a twitch interpolation technique.[19]

Physiological fatigue has been studied in patients with neuromuscular diseases,[20] in patients with chronic fatigue syndrome (CFS),[21] but also in cancer patients to evaluate cancer-related fatigue.[22] In a study of 16 cancer patients referred to palliative medicine and 16 matched non-cancer controls, neuromuscular testing was applied to determine whether cancer-related fatigue is a more centrally or peripherally mediated disorder.[22] Patients suffering from cancer-related fatigue showed less peripheral muscle fatigue and more central muscle fatigue compared to their non-fatigued controls.

It should be noted that some psychological aspects of fatigue are different for different neuromuscular diseases[23] as well as for fatigue in CFS and for cancer-related fatigue. Nevertheless, CAF appears to be a shared neurophysiological feature of fatigue in all these diseases. Therefore, it is of great interest to know whether PCF is also characterized by CAF.

Although CAF implies a central origin of fatigue, it is still unclear from which part of the central nervous system the failure originates. Therefore, further studies on brain morphology and brain metabolism are essential to elucidate the (neuro)physiological basis of fatigue.

**Brain morphology and metabolism**

Using magnetic resonance imaging (MRI), several studies have reported structural abnormalities in the brains of patients with CFS. In a group of 259 fatigued patients, specific hyperintense small punctuated subcortical white-matter foci were observed.[24] Similar results have been reported by others.[25-27] Different research groups conducted voxel-based morphometry (VBM) in CFS patients and matched healthy controls and observed gray matter volume reductions in fatigued patients compared to non-fatigued controls.[28, 29] Interestingly, it has been shown that CFS patients showed a significant increase in gray matter volume, localized in the lateral prefrontal cortex, after CBT.[30]

Magnetic resonance spectroscopy (MRS) provides a non-invasive window on metabolism in the brain. Studies with proton (^1^H) MRS allow the detection and quantification of metabolites like choline, creatine, N-acetylaspartate, and lactate. Choline is a precursor of membrane phospholipids and an elevated choline level has been associated with increased cell membrane turnover, cell density, and gliosis. Choline levels vary over the brain and the choline to creatine ratio is higher in white matter than in gray matter. In a ^1^H MRS study of the brain of CFS patients and eight age- and sex-matched healthy control subjects, the mean ratio of choline to creatine in the occipital cortex of fatigued patients was significantly higher compared to healthy controls.[31] The amount of creatine in the brain is assumed to be constant. An increased choline to creatine ratio suggests an abnormality of phospholipid metabolism and/or associated brain morphology. Similarly, in a ^1^H MRS study of the left basal ganglia, a highly significant increase in the signal from choline-containing compounds was seen in CFS patients compared to age- and sex-matched healthy controls.[32] In three children with CFS, ^1^H MRS demonstrated a significantly higher choline to creatine ratio in the frontal cortex compared to healthy controls.[33] N-acetylaspartate is considered to be a marker of neuronal density and neuronal function. In one study, reduced levels of N-acetylaspartate were observed in the right hippocampus of seven CFS patients compared to healthy controls.[34] Elevated lactate in the brain may suggest cerebral energy dysfunction. The presence of elevated ventricular lactate was observed in sixteen CFS patients as compared to healthy controls.[35]

Especially for CFS, certain characteristics of brain morphology and metabolism may serve as objective biomarkers for fatigue. However, since CFS and PCF differ in some psychological aspects[36] it still needs to be shown that these characteristics also play a role in PCF.

**Physical condition and activity**

Despite the fact that current cancer treatments are increasingly efficacious for improving survival, they are toxic in numerous ways. Therefore, many cancer patients are forced to decrease their physical activity, possibly leading to physical deconditioning. Physical activity levels were significantly reduced after diagnosis in a study of 812 breast cancer patients.[37] It may be hypothesized that compared to non-fatigued cancer survivors, PCF patients have an impaired physical condition due to decreased physical activity after cancer treatment.

In a previous study comparing physical condition in a group of 20 CFS patients and 20 matched non-fatigued sedentary controls, physical condition did not significantly differ between both groups. Although as a clinical syndrome CFS and PCF show strong similarities, in PCF a clear precipitating moment can be identified that provoked fatigue, namely the diagnosis of cancer and its subsequent treatment. In contrast to PCF, not always a precipitating factor can be identified in CFS. Therefore, studies on physical condition, specifically in cancer survivors, are needed to investigate the physiology of PCF.

**Methods/design**

**Study design**

A randomized controlled trial (RCT) with 6 months follow-up will be conducted to identify and measure (neuro)physiological factors of fatigue in severely fatigued disease-free cancer survivors and to determine the role of these factors in the maintaining of fatigue. At baseline, fatigued and non-fatigued patients will undergo measurements for peripheral and central fatigue, brain morphology and metabolism, and physical condition and activity. Fatigued patients will be randomly assigned to either the intervention group, who will immediately start CBT, or the control group, who will start CBT after six months. After six months follow-up, the measurements will be repeated in both groups of fatigued patients.

**Ethical consideration**

This study has been approved by the Medical Ethical Committee of the Radboud University Nijmegen Medical Centre (RUNMC). Patients will be informed about the study and informed consent will be obtained before randomization.

**Study population**

Patients who have been curatively treated for cancer and finished treatment at least one year before, will be asked by their physician to fill out the Checklist Individual Strength (CIS),[38] RAND-36,[39] Beck Depression Inventory for Primary Care (BDI-pc),[40] and some additional demographic and medical questions during their control-visits. Based on the scores of the fatigue severity subscale of the CIS (CIS-fatigue), patients with a cut-off score of ≥35 will be classified as severely fatigued. Severely fatigued patients, who are referred to the Expert Centre for Chronic Fatigue of the RUNMC and who met the in- and exclusion criteria of the study (Table 1), will be informed by their treating psychologist about the study. If the patients agree to be further informed about the study, the researcher will inform the patients extensively about the study and will ask for participation. Severely fatigued patients will be matched with non-fatigued patients with respect to age, sex, and previous cancer treatment (Figure 1). A total number of 57 severely fatigued and 21 non-fatigued cancer survivors will be included (see power calculation).

**Randomization and intervention**

As shown in Figure 1, severely fatigued patients will be randomized to either the intervention group (75%) or the control group (25%). Randomization will take place per patient. Patients randomized to the intervention group will be treated with CBT for PCF, as described previously.[14] The treatment consists of 12-14 individual sessions in six months. Patients randomized to the control group will be treated with CBT after six months.

**Outcome measures**

***Peripheral and central fatigue***

To obtain maximal voluntary contraction (MVC) of the biceps brachii muscle, participants will make MVCs, with 1 minute intervals, until no further force increase was observed. After a rest period of 10 minutes, peripheral and central fatigue will be measured during a 2 minute sustained MVC of the biceps brachii muscle.[18] During these 2 minutes, electrical endplate stimulation over the motor points of the medial and lateral head of the biceps brachii muscle will be applied every 15 seconds, leading to superimposed force responses. Patients will be verbally encouraged to perform maximally throughout the 2 minutes. An indicator of peripheral muscle fatigue is MFCV, which will be measured using sEMG.[16] Peripheral muscle fatigue will also be quantified by measuring the muscular force response to electrical endplate stimulation before and after the test. An indicator of central muscle fatigue is additional force production upon electrical endplate stimulation during exercise.[18] Submaximal central activation during exercise, or CAF, will be measured using a twitch interpolation technique.[19]

***Brain morphology and metabolism***

Global volumes of gray and white matter, subcortical brain volumes, and brain metabolite concentrations will be obtained. MR measurements will be performed on a 3 Tesla MR System using a radiofrequency head coil. High resolution (1mm^3^), T1 weighted MR images of the whole brain will be acquired using a magnetization prepared rapid acquisition gradient echo (MPRAGE) sequence. Normalizing, bias-correcting, and segmenting into global volumes of gray and white matter will be performed using the VBM method.[41] Automatic segmentation of subcortical brain structures was performed using the FIRST module of FSL.[42]

^1^H MR spectroscopic imaging (MRSI) will be performed, with special interest for the hippocampus, the occipital cortex, the frontal cortex, and the ventricles. The ^1^H MRSI will allow the identification and quantification of metabolites like choline, creatine, N-acetylaspartate, and lactate. Volume selection will be performed with a semi-localized by adiabatic selective refocusing (semi-LASER) pulse sequence[43] with a repetition time of 1500ms, an echo time of 30 or 136ms, and a voxel size of 10 mm^3^. The data will be analyzed using LCModel software.

***Physical condition and activity***

To measure maximal oxygen consumption (VO_2_max) a maximal exercise test will be performed.[44] In addition to the determination of VO_2_max, carbon dioxide production, heart rate, respiratory quotient, and ventilation will be measured. Patients will cycle with an increased workload of 10 Watt/minute for women and 20 Watt/minute for men. Patients will be verbally encouraged to perform maximally until exhaustion.

Daily activity will be measured during two weeks using an actometer.[45] The actometer used is a motion-sensing device, worn around the ankle, which can register and quantify human physical activity. The actometer has a piezoelectric sensor that is sensitive in three directions. Accelerations of the built-in sensor larger than a predefined threshold are considered as activity and are stored in an internal memory every five minutes. A general physical activity score expressed in the average number of accelerations per 5-min period will be provided for the whole measurement period. Actometer testing is part of the standard care if patients are referred for CBT. To assess the effect of the maximal exercise test on the daily activity of patients, they will wear an actometer for an additional five days after the maximal exercise test.

Finally, participants will score their physical activity level long before cancer diagnosis on a 10 point numeric rating scale.

***Fatigue and psychosocial measures***

Fatigued severity will be measured using the CIS-fatigue (8 items, 7-points Likert Scale, severity range from 8 to 56).[38] High scores indicate a high level of fatigue. This questionnaire has excellent psychometric properties, including good reliability and discriminative validity.[46] Simultaneously to the actometer measurements, daily observed fatigue (DOF) will be measured with the Self-Observation List (4 times a day, 4-point scale)[47] two weeks prior and five days subsequent to the maximal exercise test. This instrument gives the actual level of daily fatigue. In this way, the relation between actual fatigue levels and the (neuro)physiological measures can be accessed directly.

To examine whether self-efficacy (SE) regarding the maximal exercise test could be an explanation for possible discrepancies in VO_2_max between fatigued and non-fatigued participants, SE regarding the maximal exercise test will be measured using a task-specific SE questionnaire. This questionnaire consists of 9 questions concerning sense of control (each item scored on a 4 point numeric scale, total score range from 9 to 36) and was adapted from the self-efficacy scale (SES)[48] The SES measures the perceived level of control over fatigue, whereas the task-specific SE questionnaire used in this study measures the sense of control regarding the test. A higher score indicates a higher sense of control regarding the maximal exercise test.

To assess whether environmental factors, like social support (SS) for exercise, may influence physical activity behaviour, SS will be investigated using the shortened van Sonderen Social Support Inventory (SSL).[49] The SSL is divided into the SSL-I (amount of SS, 8 questions, score range from 8 to 32), the SSL-D (discrepancy between the amount of SS and the desired amount of SS, 8 questions, score range from 0 to 32), and the SSL-N (negative interactions, unsupportive behaviour, 7 questions, score range from 7 to 28). A higher score indicates a higher amount of SS, discrepancy, or negative interactions.

**Statistical Analysis**

Independent samples *t* tests will be performed testing baseline differences in primary and secondary outcome measures between fatigued and non-fatigued cancer survivors. Baseline differences between the therapy and waiting list condition are entered as covariates in further analyses. A paired *t* test will be performed to test whether differences can be observed within the therapy or waiting list condition from baseline to follow-up. Differences between the therapy and waiting list group on the change in outcome measures from baseline to 6 months later will be calculated with analyses of covariance (ANCOVA).

**Power calculation**

From previous studies in patients with CFS compared to healthy controls we know that groups of at least 10, preferably 20 patients per condition are sufficient to detect significant differences in (neuro)physiological parameters measured with sEMG, MRI, and exercise testing.[21, 28, 50, 51] To identify (neuro)physiological characteristics of fatigue, a comparison of baseline measurements with an age, sex, and previous cancer treatment matched group of 20 non-fatigued patients is sufficient. Fifty seven severely fatigued patients are needed to measure a change in (neuro)physiological factors due to CBT. As depicted in Figure 1, we assume that 5% of the measurements will yield technically insufficient data and 20% of the fatigued patients will drop-out from the study (14% drop-out due to failure to complete CBT or the follow-up measurements and 6% due to cancer recurrence during or after CBT or waiting list). Assuming that about two third of the patients who complete CBT will have a clinically significant response to CBT, defined as a CIS-fatigue score of less than 35 points, there will be 20 responding patients, 10 non-responding patients, and 10 untreated patients. Based on 30 patients in the CBT condition and 10 patients in the waiting list condition and assuming that the success rate of CBT is about 67% and the chance to recover spontaneously in the waiting list condition is maximally 10%, the power of the study to demonstrate a significantly greater decrease in CIS-fatigue score in patients in the intervention condition than in patients in the waiting list condition will be at least 80%.

**Discussion**

To the best of our knowledge, this will be the first study that characterizes (neuro)physiological factors of fatigue in disease-free cancer survivors and the changes of these factors in a randomized controlled way.

Studies on physiological aspects of PCF mainly concentrate on (cardiopulmonary) deconditioning. Other aspects of fatigue, like peripheral and central fatigue and brain morphology and function have been studied for patients with fatigue in the context of CFS and neuromuscular diseases and show several characteristic differences with healthy controls. CAF appears to be a shared neurophysiological feature of fatigue. CFS patients showed a significant reduced gray matter volume compared to healthy controls and even showed a significant increase in gray matter volume after CBT. Altered levels of specific metabolites in the brains of patients with CFS, measured with ^1^H MRS, have been reported. CFS patients showed a significant reduced physical activity, measured by actigraphy, compared to healthy controls. Therefore, further studies on (neuro)physiological aspects of fatigue in cancer survivors are essential not only for a theoretical understanding of this invalidating condition, but also for providing an objective biological marker that could support the diagnosis of fatigue and follow-up of the treatment of fatigue.

In conclusion, the aim of this study is to identify and measure physiological, structural, and metabolic factors of fatigue in disease-free cancer survivors and to determine the role of these factors in the maintaining of fatigue. The identification of (neuro)physiological factors of persistent fatigue can help to improve the diagnostics of fatigue, predict therapy outcome, and facilitate other treatment options. Finally, if (neuro)physiological characteristics of fatigue can be influenced by CBT, it will enhance our understanding of the mechanism causing fatigue.

**Figure**

**Figure 1** Flow-chart of the study.


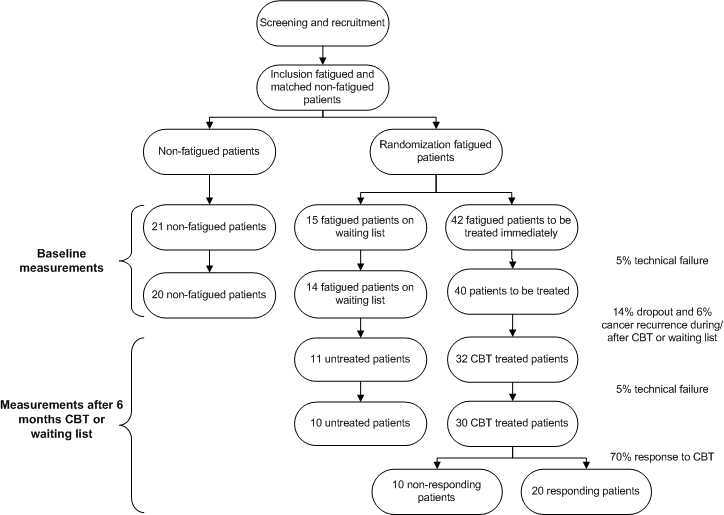


**Table**

Table 1 Inclusion and exclusion criteria

| **Inclusion criteria** |
| --- |
| 1. Age between 19 and 65 years |
| 1. Age at disease onset minimal 18 years |
| 1. Treated for a malignant, solid tumour |
| 1. Completion of treatment for cancer minimal 1 year ago   (single treatment modality surgery / current hormonal therapy permitted) |
| 1. Disease-free, as defined by the absence of somatic disease activity parameters |
| **Exclusion criteria** |
| 1. Physical comorbidity that could explain the fatigue |
| 1. Current psychological or psychiatric treatment |
| 1. Brain tumour in the past |
| 1. Contra-indication for MR examinations (e.g. claustrophobia) |
| 1. Treatment with anti-depressive drugs, anti-epileptic drugs, or benzodiazepines |
| 1. Insufficient command of the Dutch language to fill out questionnaires |

**List of abbreviations**

ANCOVA: analyses of covariance; BDI-pc: Beck depression inventory for primary care; CAF: central activation failure; CBT: cognitive behaviour therapy; CFS: chronic fatigue syndrome; CIS: checklist individual strength; CIS-fatigue fatigue severity subscale of the checklist individual strength; DOF: daily observed fatigue; ^1^H: proton; MFCV: muscle fibre conduction velocity; MPRAGE: magnetization prepared rapid acquisition gradient echo; MRI: magnetic resonance imaging; MRS: magnetic resonance spectroscopy; MRSI: magnetic resonance spectroscopic imaging; MVC: maximal voluntary contraction; PCF: postcancer fatigue; RCT: randomized controlled trial; RUNMC: Radboud University Nijmegen Medical Centre; SE: self-efficacy; sEMG: surface electromyography; semi-LASER: semi-localized by adiabatic selective refocusing; SES: self-efficacy scale; SS: social support; SSL: social support inventory; SSL-I: amount of social support; SSL-N: negative interactions of social support; SSL-D: discrepancy in social support; VBM: voxel-based morphometry.

**Competing interests**

The corresponding author and the co-authors have no conflicts of interest to declare.

**Authors’ contributions**

HP is primary investigator and is responsible for patient recruitment, data collection, data analysis, and drafting the manuscript. HL, GB, MZ, and AH designed the study and obtained funding. HL, GB, MZ, MH, and AH supervised the study. All authors read and approved the final manuscript.

**Acknowledgements**

This work is supported by the Dutch Cancer Society [KUN 2008-4002].

**References**

1. Bower JE, Ganz PA, Desmond KA, Bernaards C, Rowland JH, Meyerowitz BE, Belin TR: **Fatigue in long-term breast carcinoma survivors: a longitudinal investigation**. *Cancer* 2006, **106**(4):751-758.

2. Servaes P, van der Werf S, Prins J, Verhagen S, Bleijenberg G: **Fatigue in disease-free cancer patients compared with fatigue in patients with chronic fatigue syndrome**. *Support Care Cancer* 2001, **9**(1):11-17.

3. Irvine D, Vincent L, Graydon JE, Bubela N, Thompson L: **The prevalence and correlates of fatigue in patients receiving treatment with chemotherapy and radiotherapy. A comparison with the fatigue experienced by healthy individuals**. *Cancer Nurs* 1994, **17**(5):367-378.

4. Smets EM, Garssen B, Schuster-Uitterhoeve AL, de Haes JC: **Fatigue in cancer patients**. *Br J Cancer* 1993, **68**(2):220-224.

5. Hjermstad MJ, Fossa SD, Oldervoll L, Holte H, Jacobsen AB, Loge JH: **Fatigue in long-term Hodgkin's Disease survivors: a follow-up study**. *J Clin Oncol* 2005, **23**(27):6587-6595.

6. Nieboer P, Buijs C, Rodenhuis S, Seynaeve C, Beex LV, van der Wall E, Richel DJ, Nooij MA, Voest EE, Hupperets P *et al*: **Fatigue and relating factors in high-risk breast cancer patients treated with adjuvant standard or high-dose chemotherapy: a longitudinal study**. *J Clin Oncol* 2005, **23**(33):8296-8304.

7. Servaes P, Gielissen MF, Verhagen S, Bleijenberg G: **The course of severe fatigue in disease-free breast cancer patients: a longitudinal study**. *Psychooncology* 2007, **16**(9):787-795.

8. Servaes P, Verhagen S, Schreuder HW, Veth RP, Bleijenberg G: **Fatigue after treatment for malignant and benign bone and soft tissue tumors**. *J Pain Symptom Manage* 2003, **26**(6):1113-1122.

9. Bartsch HH, Weis J, Moser MT: **Cancer-related fatigue in patients attending oncological rehabilitation programs: prevalence, patterns and predictors**. *Onkologie* 2003, **26**(1):51-57.

10. Dimeo F, Schmittel A, Fietz T, Schwartz S, Kohler P, Boning D, Thiel E: **Physical performance, depression, immune status and fatigue in patients with hematological malignancies after treatment**. *Ann Oncol* 2004, **15**(8):1237-1242.

11. Gielissen MF, Schattenberg AV, Verhagen CA, Rinkes MJ, Bremmers ME, Bleijenberg G: **Experience of severe fatigue in long-term survivors of stem cell transplantation**. *Bone Marrow Transplant* 2007, **39**(10):595-603.

12. Okuyama T, Akechi T, Kugaya A, Okamura H, Shima Y, Maruguchi M, Hosaka T, Uchitomi Y: **Development and validation of the cancer fatigue scale: a brief, three-dimensional, self-rating scale for assessment of fatigue in cancer patients**. *J Pain Symptom Manage* 2000, **19**(1):5-14.

13. Woo B, Dibble SL, Piper BF, Keating SB, Weiss MC: **Differences in fatigue by treatment methods in women with breast cancer**. *Oncol Nurs Forum* 1998, **25**(5):915-920.

14. Gielissen MF, Verhagen S, Witjes F, Bleijenberg G: **Effects of cognitive behavior therapy in severely fatigued disease-free cancer patients compared with patients waiting for cognitive behavior therapy: a randomized controlled trial**. *J Clin Oncol* 2006, **24**(30):4882-4887.

15. Bigland-Ritchie B, Jones DA, Hosking GP, Edwards RH: **Central and peripheral fatigue in sustained maximum voluntary contractions of human quadriceps muscle**. *Clin Sci Mol Med* 1978, **54**(6):609-614.

16. Van Engelen BG, Kalkman JS, Schillings ML, Van Der Werf SP, Bleijenberg G, Zwarts MJ: **[Fatigue in neuromuscular disease]**. *Ned Tijdschr Geneeskd* 2004, **148**(27):1336-1341.

17. Zwarts MJ, Stegeman DF: **Multichannel surface EMG: basic aspects and clinical utility**. *Muscle Nerve* 2003, **28**(1):1-17.

18. Schillings ML, Hoefsloot W, Stegeman DF, Zwarts MJ: **Relative contributions of central and peripheral factors to fatigue during a maximal sustained effort**. *Eur J Appl Physiol* 2003, **90**(5-6):562-568.

19. Gandevia SC: **Neural control in human muscle fatigue: changes in muscle afferents, motoneurones and motor cortical drive [corrected]**. *Acta Physiol Scand* 1998, **162**(3):275-283.

20. Schillings ML, Kalkman JS, Janssen HM, van Engelen BG, Bleijenberg G, Zwarts MJ: **Experienced and physiological fatigue in neuromuscular disorders**. *Clin Neurophysiol* 2007, **118**(2):292-300.

21. Schillings ML, Kalkman JS, van der Werf SP, van Engelen BG, Bleijenberg G, Zwarts MJ: **Diminished central activation during maximal voluntary contraction in chronic fatigue syndrome**. *Clin Neurophysiol* 2004, **115**(11):2518-2524.

22. Yavuzsen T, Davis MP, Ranganathan VK, Walsh D, Siemionow V, Kirkova J, Khoshknabi D, Lagman R, LeGrand S, Yue GH: **Cancer-related fatigue: central or peripheral?** *J Pain Symptom Manage* 2009, **38**(4):587-596.

23. Kalkman JS, Schillings ML, van der Werf SP, Padberg GW, Zwarts MJ, van Engelen BG, Bleijenberg G: **Experienced fatigue in facioscapulohumeral dystrophy, myotonic dystrophy, and HMSN-I**. *J Neurol Neurosurg Psychiatry* 2005, **76**(10):1406-1409.

24. Buchwald D, Cheney PR, Peterson DL, Henry B, Wormsley SB, Geiger A, Ablashi DV, Salahuddin SZ, Saxinger C, Biddle R *et al*: **A chronic illness characterized by fatigue, neurologic and immunologic disorders, and active human herpesvirus type 6 infection**. *Ann Intern Med* 1992, **116**(2):103-113.

25. Greco A, Tannock C, Brostoff J, Costa DC: **Brain MR in chronic fatigue syndrome**. *AJNR Am J Neuroradiol* 1997, **18**(7):1265-1269.

26. Lange G, DeLuca J, Maldjian JA, Lee H, Tiersky LA, Natelson BH: **Brain MRI abnormalities exist in a subset of patients with chronic fatigue syndrome**. *J Neurol Sci* 1999, **171**(1):3-7.

27. Schwartz RB, Komaroff AL, Garada BM, Gleit M, Doolittle TH, Bates DW, Vasile RG, Holman BL: **SPECT imaging of the brain: comparison of findings in patients with chronic fatigue syndrome, AIDS dementia complex, and major unipolar depression**. *AJR Am J Roentgenol* 1994, **162**(4):943-951.

28. de Lange FP, Kalkman JS, Bleijenberg G, Hagoort P, van der Meer JW, Toni I: **Gray matter volume reduction in the chronic fatigue syndrome**. *Neuroimage* 2005, **26**(3):777-781.

29. Okada T, Tanaka M, Kuratsune H, Watanabe Y, Sadato N: **Mechanisms underlying fatigue: a voxel-based morphometric study of chronic fatigue syndrome**. *BMC Neurol* 2004, **4**(1):14.

30. de Lange FP, Koers A, Kalkman JS, Bleijenberg G, Hagoort P, van der Meer JW, Toni I: **Increase in prefrontal cortical volume following cognitive behavioural therapy in patients with chronic fatigue syndrome**. *Brain* 2008, **131**(Pt 8):2172-2180.

31. Puri BK, Counsell SJ, Zaman R, Main J, Collins AG, Hajnal JV, Davey NJ: **Relative increase in choline in the occipital cortex in chronic fatigue syndrome**. *Acta Psychiatr Scand* 2002, **106**(3):224-226.

32. Chaudhuri A, Condon BR, Gow JW, Brennan D, Hadley DM: **Proton magnetic resonance spectroscopy of basal ganglia in chronic fatigue syndrome**. *Neuroreport* 2003, **14**(2):225-228.

33. Tomoda A, Miike T, Yamada E, Honda H, Moroi T, Ogawa M, Ohtani Y, Morishita S: **Chronic fatigue syndrome in childhood**. *Brain Dev* 2000, **22**(1):60-64.

34. Brooks JC, Roberts N, Whitehouse G, Majeed T: **Proton magnetic resonance spectroscopy and morphometry of the hippocampus in chronic fatigue syndrome**. *Br J Radiol* 2000, **73**(875):1206-1208.

35. Mathew SJ, Mao X, Keegan KA, Levine SM, Smith EL, Heier LA, Otcheretko V, Coplan JD, Shungu DC: **Ventricular cerebrospinal fluid lactate is increased in chronic fatigue syndrome compared with generalized anxiety disorder: an in vivo 3.0 T (1)H MRS imaging study**. *NMR Biomed* 2009, **22**(3):251-258.

36. Servaes P, Prins J, Verhagen S, Bleijenberg G: **Fatigue after breast cancer and in chronic fatigue syndrome: similarities and differences**. *J Psychosom Res* 2002, **52**(6):453-459.

37. Irwin ML, Crumley D, McTiernan A, Bernstein L, Baumgartner R, Gilliland FD, Kriska A, Ballard-Barbash R: **Physical activity levels before and after a diagnosis of breast carcinoma: the Health, Eating, Activity, and Lifestyle (HEAL) study**. *Cancer* 2003, **97**(7):1746-1757.

38. Vercoulen JH, Swanink CM, Fennis JF, Galama JM, van der Meer JW, Bleijenberg G: **Dimensional assessment of chronic fatigue syndrome**. *J Psychosom Res* 1994, **38**(5):383-392.

39. VanderZee KI, Sanderman R, Heyink JW, de Haes H: **Psychometric qualities of the RAND 36-Item Health Survey 1.0: a multidimensional measure of general health status**. *Int J Behav Med* 1996, **3**(2):104-122.

40. Beck AT, Guth D, Steer RA, Ball R: **Screening for major depression disorders in medical inpatients with the Beck Depression Inventory for Primary Care**. *Behav Res Ther* 1997, **35**(8):785-791.

41. Ashburner J, Friston KJ: **Voxel-based morphometry--the methods**. *Neuroimage* 2000, **11**(6 Pt 1):805-821.

42. Patenaude B, Smith SM, Kennedy DN, Jenkinson M: **A Bayesian model of shape and appearance for subcortical brain segmentation**. *Neuroimage* 2011, **56**(3):907-922.

43. Scheenen TW, Klomp DW, Wijnen JP, Heerschap A: **Short echo time 1H-MRSI of the human brain at 3T with minimal chemical shift displacement errors using adiabatic refocusing pulses**. *Magn Reson Med* 2008, **59**(1):1-6.

44. Cox NJ, Hendriks JC, Binkhorst RA, Folgering HT, van Herwaarden CL: **Reproducibility of incremental maximal cycle ergometer tests in patients with mild to moderate obstructive lung diseases**. *Lung* 1989, **167**(2):129-133.

45. Vercoulen JH, Bazelmans E, Swanink CM, Fennis JF, Galama JM, Jongen PJ, Hommes O, Van der Meer JW, Bleijenberg G: **Physical activity in chronic fatigue syndrome: assessment and its role in fatigue**. *J Psychiatr Res* 1997, **31**(6):661-673.

46. Vercoulen JH, Swanink CM, Galama JM, Fennis JF, Jongen PJ, Hommes OR, van der Meer JW, Bleijenberg G: **The persistence of fatigue in chronic fatigue syndrome and multiple sclerosis: development of a model**. *J Psychosom Res* 1998, **45**(6):507-517.

47. Bleijenberg G, Kuijpers HC: **Biofeedback treatment of constipation: a comparison of two methods**. *Am J Gastroenterol* 1994, **89**(7):1021-1026.

48. Wiborg JF, Knoop H, Frank LE, Bleijenberg G: **Towards an evidence-based treatment model for cognitive behavioral interventions focusing on chronic fatigue syndrome**. *J Psychosom Res* 2012, **72**(5):399-404.

49. van Sonderen E: **Het meten van sociale steun met de Sociale Steun Lijst - Interacties (SSL-I) en Sociale Steun Lijst - Discrepancies (SSL-D), een handleiding.** Groningen: Noorderlijk Centrum voor Gezondheidsvraagstukken; 1993.

50. Bazelmans E, Bleijenberg G, Van Der Meer JW, Folgering H: **Is physical deconditioning a perpetuating factor in chronic fatigue syndrome? A controlled study on maximal exercise performance and relations with fatigue, impairment and physical activity**. *Psychol Med* 2001, **31**(1):107-114.

51. Bazelmans E, Bleijenberg G, Voeten MJ, van der Meer JW, Folgering H: **Impact of a maximal exercise test on symptoms and activity in chronic fatigue syndrome**. *J Psychosom Res* 2005, **59**(4):201-208.
